# Novel Natural Inhibitors Targeting Enhancer of Zeste Homolog 2: A Comprehensive Structural Biology Research

**DOI:** 10.3389/fonc.2021.741403

**Published:** 2021-10-19

**Authors:** Weihang Li, Ziyi Ding, Yunlong Zhao, Min Jiang, Shilei Zhang, Hongzhe Zhao, Ke Lei, Rui Xu, Yingjing Zhao, Dong Wang, Min Chao, Yanjiang Yin, Changbin Yang, Liang Wang, Ming Yan

**Affiliations:** ^1^ Department of Orthopedic Surgery, Xijing Hospital, The Fourth Military Medical University, Xi’an, China; ^2^ College of Clinical Medicine, China-Japan Union Hospital of Jilin University, Changchun, China; ^3^ Department of General Surgery, Zhen an County People’s Hospital, Shangluo, China; ^4^ State Key Laboratory of Oncology in South China, Collaborative Innovation Center for Cancer Medicine, Sun Yat-sen University Cancer Center, Guangzhou, China; ^5^ Department of Endocrinology, Shanghai National Research Center for Endocrine and Metabolic Disease, State Key Laboratory of Medical Genomics, Shanghai Institute for Endocrine and Metabolic Disease, Ruijin Hospital, Shanghai Jiaotong University School of Medicine, Shanghai, China; ^6^ Department of Intensive Care Unit, Nanjing First Hospital, Nanjing Medical University, Nanjing, China; ^7^ Department of Neurosurgery, Tangdu Hospital of Fourth Military Medical University, Xi’an, China; ^8^ Department of Hepatobiliary Surgery, National Cancer Center/National Clinical Research Center for Cancer/Cancer Hospital, Chinese Academy of Medical Sciences and Peking Union Medical College, Beijing, China; ^9^ Military Medical Innovation Center, The Fourth Military Medical University, Xi’an, China

**Keywords:** EZH2, inhibitor, histone methyltransferase, structural biology, virtual screening

## Abstract

The enhancer of zeste homolog 2 (EZH2) is a methylated modification enzyme of Histone H3-Lys 27. The high expression of EZH2 in cells is closely related to the progression, invasion, and metastasis of neoplasm. Therefore, this target is gradually becoming one of the research hot spots of tumor pathogenesis, and the inhibitors of the EZH2 enzyme are expected to become new antitumor drugs. This study used a series of virtual screening technologies to calculate the affinity between the compounds obtained from the ZINC15 database and the target protein EZH2, the stability of the ligand–receptor complex. This experiment also predicted the toxicity and absorption, distribution, metabolism, and excretion (ADME) properties of the candidate drugs in order to obtain compounds with excellent pharmacological properties. Finally, the ligand–receptor complex under *in vivo* situation was estimated by molecular dynamics simulation to observe whether the complex could exist steadily in the body. The experimental results showed that the two natural compounds ZINC000004217536 and ZINC000003938642 could bind tightly to EZH2, and the ligand–receptor complex could exist stably *in vivo*. Moreover, these two compounds were calculated to be nontoxic. They also had a high degree of intestinal absorption and high bioavailability. *In vitro* experiments confirmed that drug ZINC000003938642 could inhibit the proliferation and migration of osteosarcoma, which could serve as potential lead compounds. Therefore, the discovery of these two natural products had broad prospects in the development of EZH2 inhibitors, providing new clues for the treatment or adjuvant treatment of tumors.

## Introduction

EZH2, namely, enhancer of zeste homolog 2, is a pivotal member of epigenetic regulatory factor Polycomb group (PcG) proteins. PcG proteins can lead to gene suppression through methylation modification (1), which comprises several essential molecules like Polycomb repressive complexes (PRCs). PRCs have inherent histone methyltransferase (HMTase) activity, which can inhibit gene expression through core histone methylation (2). PRC2 is of vital importance in PcG proteins, as it plays a role in the development of cancer (3). PRC2 consists of three subunits: EZH2, SUZ12, and EED, of which EZH2 and chaperone proteins are essential to correctly coordinate differentiation and proliferation of cells (4).

EZH2 has methyltransferase activity and can catalyze the methylation of histone H3-Lys 27 (H3-K27); it is essential for PRC-mediated gene suppression (5). Research had reported that human EZH2 was upregulated in different kinds of tumors like breast cancer, prostate cancer, and osteosarcoma (OS) (6). Cyclin-dependent kinase 1 (CDK1) promotes EZH2 ubiquitination by mediating the phosphorylation of Thr-345 and Thr-487 (T345 and T487) sites of EZH2 (7). And the posttranslational modifications of EZH2 are essential to improve its protein stability that related to the function of tumor cells and tumor metastasis, which could further lead to the accumulation of EZH2 and the occurrence of cancers (8, 9).

In summary, EZH2 is related to different kinds of neoplasms, which was abnormally expressed and could serve as a therapeutic target (10–12). Therefore, inhibition of EZH2 protein could provide new ideas and methods in the treatment of cancers. GSK126 is a new type of competitive inhibitor targeting EZH2, which had begun tests in clinical trials (13). GSK126 significantly reduces the level of H3K27me3 in tumor cells by inhibiting the methyltransferase activity of EZH2, thereby inhibiting the growth of tumor cells such as human tongue squamous cell carcinoma and multiple myeloma cells (14, 15). In addition, the appropriate concentration of GSK126 could also induce tumor cell apoptosis through the mitochondrial pathway (16). Research also reported that EZH2 may promote tumor invasion and metastasis by downregulating downstream targets such as E-cadherin and vascular endothelial growth factor (VEGF)-A (17, 18). VEGF-A is an important cytokine that regulates angiogenesis, which is closely related to tumor metastasis (19). Research on EZH2 inhibitors has become hot spots in recent years, which has changed the treatment scheme as well as ideals dramatically. Nevertheless, novel efficient inhibitors targeting EZH2 still remained less. Consequently, more inhibitors regarding EZH2 were needed to discover from a natural medicine library in order to screen novel natural lead compounds and provide new clues in the discovery of EZH2 inhibitors. Existing studies had confirmed that EZH2 was highly expressed in OS patients and could serve as potential biomarker (11), while research on targeted therapy of OS targeting EZH2 had hardly been reported. Up to now, the research on EZH2 inhibitor GSK126 had made notable progress in different kinds of cancers, including prostate cancer cells and gastric cancer cells (20, 21). Consequently, this study chose GSK126 as the reference compound to compare the pharmacological properties of the candidate compounds in order to discover more potential lead compounds targeting EZH2. Besides, this study aimed to validate whether EZH2 could serve as a therapeutic target in the treatment of OS.

Recently, natural products and natural extracts may be highly available compounds with proper biological activity that has potential medicinal value. They are therefore important sources for discovering, designing, and improving new drug skeletons (22, 23). Extensive investigations have shown that natural products and their derivatives are currently playing an important role in the medical industry. It is already determined that the natural compounds from natural product database have considerable pharmacological potential (24, 25). The second part of this study provides a theoretical basis and guidelines for discovering new inhibitors from natural product repository by screening inhibitory compounds related to EZH2. Besides, absorption, distribution, metabolism, and excretion (ADME) and toxicity prediction, ligand binding research, and molecular dynamics (MD) simulation were carried out on the selected candidate compounds, laying the foundation for the improvement of tumor drugs.

## Materials and Methods

### Discovery Studio Software and Ligand Library

The LibDock module of Discovery Studio 4.5 software (BIOVIA, San Diego, CA, USA) is used to screen better energy-optimized natural products, and the ADMET module can be applied to ADME analysis and the prediction of carcinogenicity and Ames mutagenicity. The CDOCKER module can be used to analyze the binding force between the products and the corresponding target of the protein and analyze the stability of the complex. This experiment selects the natural product database in the ZINC database to screen EZH2 inhibitors. The ZINC15 database is provided by the Irwin and Shoichet Laboratories in the Department of Pharmaceutical Chemistry at the University of California, San Francisco (UCSF), which provides a free virtual screening database of commercially available compounds.

### Structure-Based Virtual Screening Using LibDock

The LibDock module was widely used in the drug development process (26). The LibDock module used a grid placed in the binding site and used polar and non-polar probes to calculate protein hot spots, then further used hot spots to arrange the ligands to form favorable interactions. Moreover, the study also used the Smart Minimiser algorithm and the CHARMm force field (Cambridge, MA, USA) to minimize the ligands (27). Then, all ligands’ positions were adjusted and ranked according to the calculated ligand scores. The 2.5-Å crystal structure of human EZH2 [Protein Data Bank (PDB) identifier: 5WF7] and the structure of the inhibitor GSK126 were downloaded respectively from the PDB and ZINC15 database, and they were imported into the working environment of LibDock. **Figure 5** shows the chemical structure of EZH2. The protein was prepared through several steps, including removing the crystal water and other surrounding heteroatoms, then adding hydrogen, protonation, ionization, and energy minimization. Among them, the energy minimization was realized by the CHARMM force field and the Smart Minimiser algorithm. In the case that the root mean square gradient tolerance was 0.1, the minimization performed 2,000 steps. After calculation, the binding site of the prepared protein was defined through the “Edit binding site” option. Analyzing the binding site of the ligand (GSK126) to generate the active binding site for docking the ligand with the receptor. Virtual screening was performed by docking the ligand exported from the database with the defined active binding site through the LibDock module. All compounds were grouped and ranked according to their LibDock score.

### Prediction of Absorption, Distribution, Metabolism, and Excretion and Toxicity

The ADMET module of DS4.5 was used to estimate the adsorption, distribution, metabolism, and excretion properties of compounds. And the TOPKAT module of DS4.5 was employed to predict the carcinogenicity, Ames mutagenicity, and developmental toxicity potential in rodents. These pharmacological properties are fully considered when screening suitable EZH2 inhibitors to ensure the safety of the drug.

### Molecule Docking and the Prediction of Drug Affinity

The CDOCKER module of DS4.5 was used for molecular docking research. CDOCKER is a tool to calculate high-precision docking results based on the CHARMM force field. During the docking process, the structures of the ligands are allowed to bend, while the structure of the receptor remains rigid. The CHARMM energy and interaction energy of each posture generated are calculated to reflect the binding affinity of the ligand and the receptor. Since crystal water molecules may affect the formation of receptor–ligand complexes, fixed water molecules are usually removed during the semi-flexible and rigid docking process, and hydrogen atoms are added to the protein to ensure the accuracy of the experiment (28, 29). The crystal structure of EZH2 was obtained from the protein database, and the three-dimensional structures of ZINC3938642 and ZINC4217536 were obtained from the ZINC15 database. In order to verify the reliability of the results, this experiment also downloaded the reference compound GSK126 from the ZINC15 database. Similarly, the GSK126 was docked with EZH2 to calculate the root mean square deviation (RMSD) of the molecular docking conformation and compared it with the RMSD of the conformations of the ligand–receptor complex that are selected in this experiment. The binding site of EZH2 is defined as an area within a 5-Å radius from the geometric center of the ligand GSK126. In this experiment, the selected ligand was allowed to bind to the protein group residues in the binding site sphere. The identified hit structures were prepared and docked with the binding site of EZH2. Based on the numerical values of CDOCKER interaction energy, the different postures of each ligand–EZH2 receptor complex were generated and analyzed in detail.

### Molecular Dynamics Simulation

Among the various postures predicted by the molecular docking program, the best binding conformation of the EZH2–inhibitor complex is selected as the object for MD simulation. The ligand–receptor complex is placed in an orthogonal box and solved with an explicit periodic boundary solvated water model. At the same time, to simulate the physiological environment, sodium chloride with an ionic strength of 0.145 was added to the system. Then, the system is subjected to the CHARMM force field and is relaxed through energy minimization (1,000 steps of steepest descent and 1,000 steps of the conjugated gradient). The reaction system was slowly driven from the initial temperature of 50K to the target temperature of 300K, the driving time was 2 ns, and the equilibrium simulation was performed when the time was 1 ns. The time for MD simulation (production) is 40 ns, and the time step is 2 fs. The simulation adopts the NPT (normal pressure and normal temperature) system at a constant temperature close to 300K, and the results were stored at a frequency of 0.02 ns. The Particle Mesh Ewald algorithm was used to calculate the long-range static electricity, and the linear constraint solver algorithm was used to fix all bonds involving hydrogen. Set the initial complex as the reference object. Use the Discovery Studio 4.5 analyze trajectory protocol to determine the structural properties, RMSD, and potential energy of the trajectory simulated by MD.

### Cell Lines and Reagents

Human OS cell lines MG-63 (CL-0157), HOS (CL-0360), and human normal liver cell line LO2 (CL-0111) were purchased from Procell Life Science & Technology Co., Ltd. These cell lines were incubated in high-glucose Dulbecco’s modified Eagle’s medium (DMEM; Procell, Cat. no. PM150210), containing 10% fetal bovine serum (FBS; Gemini, USA) and 100 units/ml penicillin and 100 mg/ml streptomycin under normal cell culture conditions (37°C and 5% CO_2_). Drug ZINC000003938642 was provided by Selleck Chemical Co. (Cat. no. S3668). The drug was dissolved in ultrapure water based on manufacturer-provided instructions to obtain the stock solution. Dimethylsulfoxide (DMSO) was not used to dissolve the drug in this study so that the toxicity effect on cells was negligible. Then, appropriate culture medium was added into the stock solution to configure different concentrations of the drug.

### Cell Counting Kit-8 Assay

The standard Cell Counting Kit-8 (CCK-8) assay (provided by ApexBio, USA) was conducted to measure the cellular viability and proliferation of OS cells (HOS and MG-63) and human liver cell (LO2). Cell lines were plated into 96-well culture plates with a density of 3,000 cells/well overnight. Cells were treated with different doses of drug ZINC000003938642 for 24 h. The concentration gradients of each treatment were 0, 5, 10, 20, 40, 80, 160, 320, and 540 μmol/L. Cells were cultured for 1.5 h after addition of 10 μl/well CCK-8, and then the OD value of each well was measured based on the wavelength of 450 nm according to the microplate reader (BioTek Instrument, Synergy H1, USA).

### Colony Formation Assay

Colony formation assay (CFA) assay was performed to detect the effects of different doses of drug on proliferation of tumor cells. HOS and MG-63 cells were incubated into six-well plate with the density of 600 cells/well. After 24 h in culture, we configured cell culture medium with drug concentration of 100, 250 μmol/L; DMSO was not used in this study so the influence of DMSO on cells could be neglected. After 10 days of cultivation, the developed colonies were rinsed with phosphate buffered saline (PBS) twice and fixed in 4% paraformaldehyde, then 0.5% crystal violet solution was used to stain the colonies for half an hour. Lastly, we counted and described colonies according to microscopic examination.

### *In Vitro* Scratch Assay

OS cells (HOS and MG-63) were cultured in six-well plate to assess the effects of drug on the migration of tumor cells. When the degree of fusion reached 90%, a 1-ml pipette tip was used to make a consistent cell-free area. Then, PBS was used to rinse twice to wipe off the cell debris, and serum-free medium was changed to culture, and different concentrations (0, 25, 50, 100, 250 μmol/L) of drug were used to treat cells and observe the scratch width at 0, 6, 12, 24 h. After corresponding time, we captured images of scraped area with phase contrast microscopy and measured the wounds and scratch width. The migration rate of OS cells was calculated as:


$$\text{percentage of wound closure}=\frac{(scratch\;area\;of\;0H-scratch\;area\;of\;corresponding\;time)\;}{scratch\;area\;of\;0H}$$

### Western Blotting

OS cell lines (HOS and MG-63) were seeded into T25 culture flask and treated with different doses of drug ZINC000003938642 for 48 h. Then, proteins were extracted by radioimmunoprecipitation assay (RIPA), and bicinchoninic acid (BCA) protein assay was conducted to define protein standard curve and detect the protein concentration of each sample. Ten percent sodium dodecyl sulfate-polyacrylamide gel electrophoresis (SDS-PAGE) was used to separate proteins of samples, and then proteins were transferred to polyvinylidene difluoride (PVDF) membranes. Five percent nonfat milk dissolved in Tris-buffered saline and tween 20 (TBST) buffer was used to block the membranes for 2 h, after that, the membranes were incubated with primary antibodies [EZH2, c-Myc from Abcam and glyceraldehyde 3-phosphate dehydrogenase (GAPDH) from Proteintech] at 4°C overnight. On the second day, the membranes were washed with Tris-buffered saline and tween 20 (TBST) three times and then horseradish peroxidase-conjugated secondary antibody was added to incubate the membranes for 1 h at room temperature. The membranes were visualized with enhanced chemiluminescence reagents to detect corresponding protein signals. Viber Bio Imaging tools were used to measure the band densities.

### Apoptosis Assay

OS cells (HOS and MG-63) in log growth phase were inoculated into six-well plate and were treated with different concentrations of the drugs. After culturing for 24 h, cells were extracted through trypsin (without EDTA) and Annexin-fluorescein isothiocyanate (FITC)/propidium iodide (PI) double staining was performed according to the manufacturer’s instructions. Lastly, the stained cells were analyzed by flow cytometry techniques; the apoptosis rates were examined by ACEA NovoCyte flow cytometry.

### Pharmacophore Predictions of the Ideal Lead Compounds

After initial validation of the antitumor effects of the selected compounds, this study further analyzed their pharmacophore characteristics. Pharmacophore predictions of compounds were performed according to 3D-QSAR pharmacophore generation module, which generated up to 255 fits per molecule to represent a small molecule, and only fits with energy values within the energy threshold of 10 kcal/mol were finally preserved.

## Results

### EZH2 Expression in Third-Party Database

**Figure 1:**
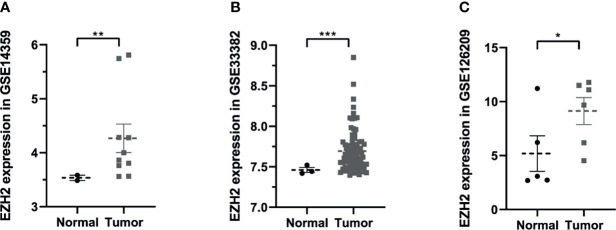
The expression situation of Enhancer of Zeste Homolog 2 (EZH2) between osteosarcoma and normal patients in Gene Expression Omnibus (GEO) database: **(A)** GSE14359, **(B)** GSE33382, **(C)** GSE126209. Data were represented as mean ± SEM. *P < 0.05; **P < 0.01; ***P < 0.0001; the same below.

To figure out the expression situation of EZH2 in OS, this study analyzed the expression values of EZH2 between normal and OS patients in Gene Expression Omnibus (GEO, https://www.ncbi.nlm.nih.gov/geo/) database. In total, three GSE series were analyzed including GSE14359, GSE33382, and GSE126209. As shown in **Figure 1**, results demonstrated that the expression of EZH2 in OS patients was significantly upregulated compared with that in normal patients (P < 0.05, Wilcoxon nonparametric test).

### Fast Virtual Screening of Potential Inhibitors of EZH2

**Figure 2:**
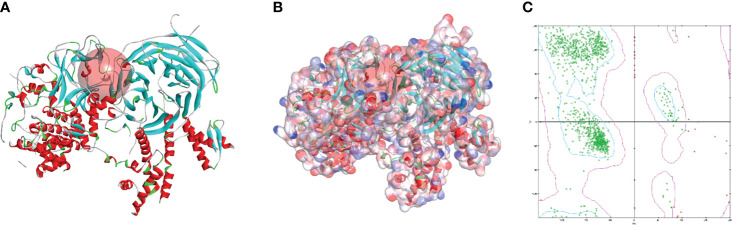
The molecular structure of Enhancer of Zeste Homolog 2 (EZH2). **(A)** Initial molecule structure and added active binding sphere, the active binding sphere was shown as red region. **(B)** Surface of binding region added. Blue represented positive charge, and red represented negative charge. **(C)** The Ramachandran diagrams of EZH2 protein.

The SAL/SET domain of EZH2 protein is regarded as an important regulatory site for its enzymatic activity. Inhibitors bind to the SAL/SET domain of EZH2 by inserting into the ligand pocket of EZH2 and exerts the function of inhibiting the activity of EZH2: The small molecules binding to this site can prevent S-adenosyl methionine (SAM) from providing EZH2 with the methyl group needed to methylate H3K27me3, thereby reducing the enzymatic activity of EZH2. After SAM loses its methyl group, it is metabolized and hydrolyzed into intermediate products including S-adenosyl-L-homocysteine (SAH) and adenosine. S-adenosylmethionine is a methyl donor for one-carbon unit metabolism in organisms, and by moderately promoting the metabolic level of SAM, the activity of EZH2 can be inhibited (30). Based on this mechanism, inhibitors of enzyme activity against EZH2 could be identified. Therefore, this domain was chosen as the docking site for screening. The crystal structure of EZH2 was displayed in **Figure 2**, which contained the binding site sphere for docking, as well as the Ramachandran diagram of the protein, to check the rationality of EZH2 structure. Firstly, LibDock module of DS4.5 was performed to virtually screen small molecules that functioned in binding with the receptor protein EZH2. Downloading commercially available natural compounds from the ZINC15 database, a total of 13,537 ligands were generated by virtual screening. At the same time, the effective selective inhibitor GSK126, which could inhibit the activity of EZH2, was selected as the reference compound. After screening, 669 compounds were found with higher LibDock scores than GSK126 (LibDock score: 132.143). The top 20 compounds were listed in **Table 1**.

**Table 1:** Top 20 ranked compounds with higher Libdock scores than GSK126.

Number	Compounds	Libdock Score
1	ZINC000085545908	207.393
2	ZINC000085544839	207.175
3	ZINC000004096059	198.67
4	ZINC000004099069	194.59
5	ZINC000008552069	193.20
6	ZINC000056897657	191.551
7	ZINC000004217536	191.439
8	ZINC000095620524	189.085
9	ZINC000004096684	187.339
10	ZINC000062238222	184.582
11	ZINC000100084136	183.874
12	ZINC000150338786	182.677
13	ZINC000014951658	182.425
14	ZINC000003938642	181.651
15	ZINC000004096878	181.45
16	ZINC000004099068	180.745
17	ZINC000004096653	180.432
18	ZINC000085826837	178.464
19	ZINC000049784088	178.376
20	ZINC000008220033	177.227

### Absorption, Distribution, Metabolism, and Excretion Characteristics and Toxicity Prediction

By using the ADME and TOPKAT prediction module, we obtained the candidate 20 kinds of ligands and GSK126’s pharmacological properties, including penetration of the blood–brain barrier, degree of human intestinal absorption, water solubility level, inhibitory effect on cytochrome P450 2D6, hepatotoxicity, and plasma protein binding properties (**Table 2**). The water solubility prediction showed that 18 compounds could be dissolved in water relatively well. Among them, 10 compounds had a high solubility level (scores >2), which were greater than the reference compound GSK126 (moderate solubility, score: 1). For the degree of human intestinal absorption, 19 compounds had a good absorption effect, the same as GSK126, and ZINC000085826837 had a medium absorption level. Besides, seven compounds and GSK126 could be bound strongly by plasma proteins, while the remaining compounds did not have tight binding affinity and strong interactions with plasma proteins. Cytochrome P4502D6 (CYP2D6) was a key enzyme in the process of drug metabolism. Compounds involved in the screening had no inhibitory effect on CYP2D6. GSK126 was also predicted to be a non-inhibitor of CYP2D6. For liver toxicity, 12 compounds were predicted to be nontoxic drugs, while the remaining compounds and GSK126 were toxic to the liver.

**Table 2:** Adsorption, distribution, metabolism, and excretion (ADME) properties of compounds.

Number	Compound	Solubility level	BBB level	CYP2D6	Hepatotoxicity	Absorption level
1	ZINC000085545908	3	4	FALSE	FALSE	3
2	ZINC000085544839	3	4	FALSE	TRUE	3
3	ZINC000004096059	1	4	FALSE	TRUE	3
4	ZINC000004099069	3	4	FALSE	FALSE	3
5	ZINC000056897657	1	4	FALSE	TRUE	3
6	ZINC000004217536	3	4	FALSE	FALSE	3
7	ZINC000095620524	4	4	FALSE	TRUE	3
8	ZINC000008552069	4	4	FALSE	TRUE	3
9	ZINC000004096684	1	4	FALSE	FALSE	3
10	ZINC000062238222	3	4	FALSE	TRUE	3
11	ZINC000100084136	1	4	FALSE	FALSE	3
12	ZINC000150338786	1	4	FALSE	TRUE	3
13	ZINC000014951658	3	4	FALSE	FALSE	3
14	ZINC000003938642	0	4	FALSE	FALSE	3
15	ZINC000004096878	1	4	FALSE	TRUE	3
16	ZINC000004099068	3	4	FALSE	FALSE	3
17	ZINC000004096653	1	4	FALSE	FALSE	3
18	ZINC000085826837	2	4	FALSE	FALSE	2
19	ZINC000049784088	4	4	FALSE	FALSE	3
20	ZINC000008220033	0	4	FALSE	FALSE	3
21	Reference ligand	1	4	FALSE	TRUE	3

**Figure 3:**
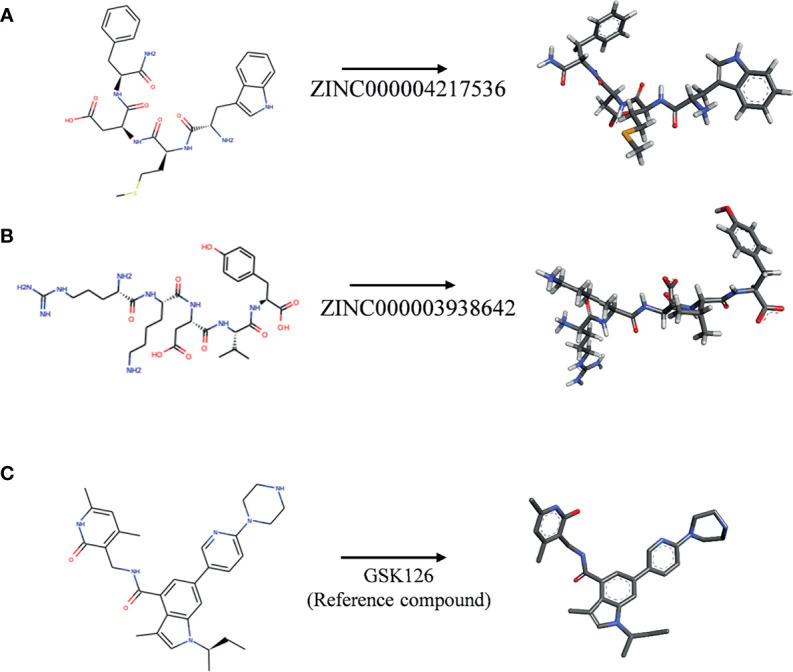
The 2D structures of novel compounds selected from virtual screening and the reference compound GSK126 by chemdraw. **(A)** ZINC000004217536, **(B)** ZINC000003938642, and **(C)** GSK126.

Subsequently, this experiment also calculated the safety properties of the candidate compounds and GSK126 through the TOPKAT module, including Ames (Ames mutagenicity), developmental toxicity potential (DTP), and rodent carcinogenicity [based on the United States National Toxicology Program (NTP) data set]. Experimental results displayed that those 12 compounds were non-mutagenic in long-term effect. It was predicted that four compounds were non-carcinogens and three compounds had no developmental toxicity potential. In addition, the reference compound GSK126 also predicted with pretty characteristics on Ames and NTP carcinogenicity, while it was computed with probability of DTP. The detailed information of the indicators among compounds and GSK126 were shown in **Table 3**. Based on the above data, ZINC000004217536 and ZINC000003938642 were neither CYP2D6 inhibitors nor hepatotoxicity drugs. Moreover, they were predicted to be free of Ames mutagenicity and rodent carcinogenicity. Consequently, ZINC000004217536 and ZINC000003938642 were analyzed to be candidate drugs with high safety and were selected for further study. The detailed chemical and crystal structures of these compounds were shown in **Figure 3**.

**Table 3:** Toxicities of compounds.

Number	Compounds	NTP: Mouse		NTP: Rat		Ames	DTP
		Female	Male	Female	Male		
1	ZINC000085545908	1	0	0	0	0	1
2	ZINC000085544839	0	0.851	0	0.954	0.001	1
3	ZINC000004096059	0	0	0	1	0	1
4	ZINC000004099069	0	0	0	0	0.002	0.846
5	ZINC000056897657	0	0.982	1	0	1	1
6	ZINC000004217536	0	0	0	0	0	1
7	ZINC000095620524	1	0	1	0.999	0	1
8	ZINC000008552069	0.015	0	0	0.997	1	1
9	ZINC000004096684	0	1	1	0	1	1
10	ZINC000062238222	0	0	0	0.969	0.989	1
11	ZINC000100084136	0	0	0	1	0	0
12	ZINC000150338786	0	1	0	0	1	1
13	ZINC000014951658	1	0	1	0	0	1
14	ZINC000003938642	0	0	0	0	0	0
15	ZINC000004096878	0	1	0	0	1	1
16	ZINC000004099068	0	0	0	0	0.002	0.864
17	ZINC000004096653	0	1	1	0	1	1
18	ZINC000085826837	0.186	1	1	0.998	0	1
19	ZINC000049784088	0.995	0	0	0.008	1	1
20	ZINC000008220033	0	1	1	0	0	0
21	GSK126	1.000	0.003	0.000	0.340	0.009	0.924

NTP, National Toxicology Program.

### Ligand–EZH2 Binding Analysis

**Figure 4:**
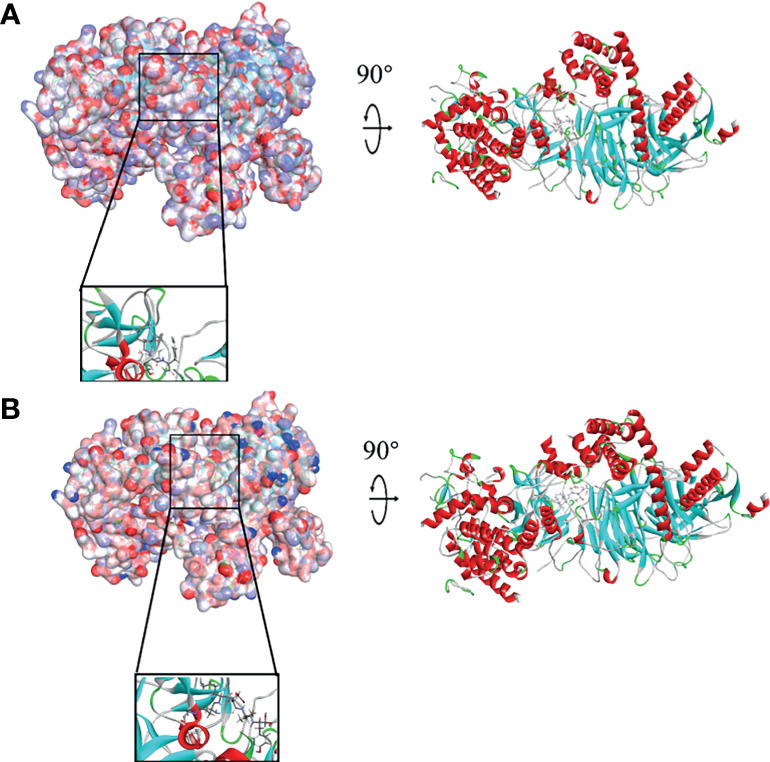
Schematic drawing of interactions between ligands and Enhancer of Zeste Homolog 2 (EZH2). The surface of binding area was added; blue represented positive charge, red represented negative charge; and ligands were shown in sticks; the structures around the ligand–receptor junction were shown in thinner sticks. **(A)** ZINC000004217536–EZH2 complex. **(B)** ZINC000003938642–EZH2 complex.

**Figure 5:**
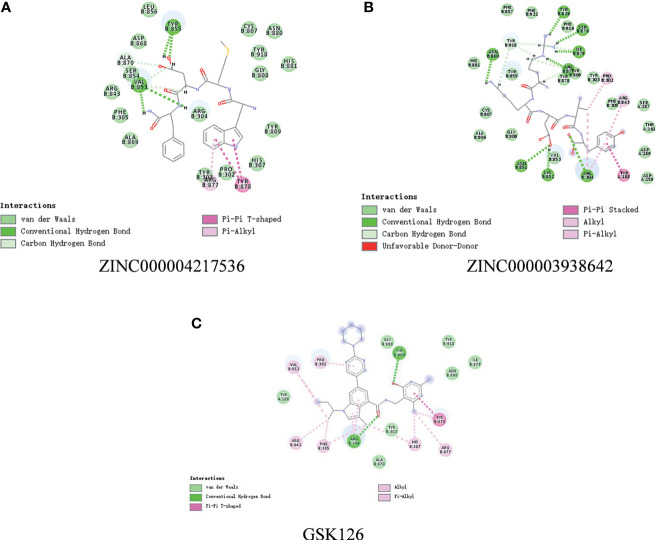
The detailed intermolecular interaction of the predicted binding modes of **(A)** ZINC000004217536, **(B)** ZINC000003938642, and **(C)** GSK126 to Enhancer of Zeste Homolog 2 (EZH2).

In order to study the binding mechanisms between the ligand and receptor EZH2, CDOCKER module was conducted to dock ZINC000004217536, ZINC000003938642, and GSK126 at the regulatory site of EZH2, and the corresponding CDOCKER potential energy of these complexes was calculated, as shown in **Table 4**. Hydrogen bonds and π–π interactions between EZH2 and these compounds were analyzed (**Figures 4**, **5**). Results visualized that ZINC000004217536 formed four pairs of hydrogen bonds with EZH2: O27 with TYR855:HN of EZH2, H68 with TYR855:O of EZH2, and H70 and H79 with VAL853:O of EZH2. ZINC000003938642 formed eight pairs of hydrogen bonds with EZH2: H62 with ARG304:NH1 of EZH2, H62 with LYS852:O of EZH2, O11 with ASN851:ND2 of EZH2, H74 with ASN880:OD1 of EZH2, H89 with TYR826:OH of EZH2, H90 with SER876:O of EZH2, H91 with ILE879:O of EZH2, and H86 with ARG877:O of EZH2. Two pairs of hydrogen bonds were formed between the reference compound GSK126 and EZH2: O12 with ARG304:N of EZH2 and O23 with TYR809:N of EZH2. Additionally, these three compounds formed two pairs, one pair each of π–π interactions with EZH2. The detailed chemical bond interactions were displayed in **Table 5**.

**Table 4:** CDOCKER interaction energy of selected compounds with Enhancer of Zeste Homolog 2 (EZH2).

Compound	CDOCKER potential energy (Kcal/mol)
ZINC000004217536	-58.3934
ZINC000003938642	-52.6615
GSK126	-46.7202

**Table 5:** Hydrogen Bond Interaction Parameters for Each Compound with EZH2.

Receptor	Compound	Donor Atom	Receptor Atom	Distances (Å)
EZH2	ZINC000004217536	B:TYR855:HN	ZINC000004217536:O27	2.02
		ZINC000004217536:H68	B:TYR855:O	3.10
		ZINC000004217536:H70	B:VAL853:O	2.76
		ZINC000004217536:H79	B:VAL853:O	2.46
	ZINC000003938642	B:ARG304:NH1	ZINC000003938642:O47	3.05
		ZINC000003938642:H62	B:LYS852:O	2.27
		B:ASN851:ND2	ZINC000003938642:O11	3.39
		ZINC000003938642:H74	B:ASN880:OD1	2.19
		ZINC000003938642:H89	B:TYR826:OH	2.24
		ZINC000003938642:H90	B:SER876:O	2.38
		ZINC000003938642:H91	B:ILE879:O	2.05
		ZINC000003938642:H86	B:ARG877:O	1.89
	GSK126	B:ARG304:N	K:A9G8009:O12	3.00
		B:TYR809:N	K:A9G8009:O23	2.89

### Molecular Dynamics Simulation

**Figure 6:**
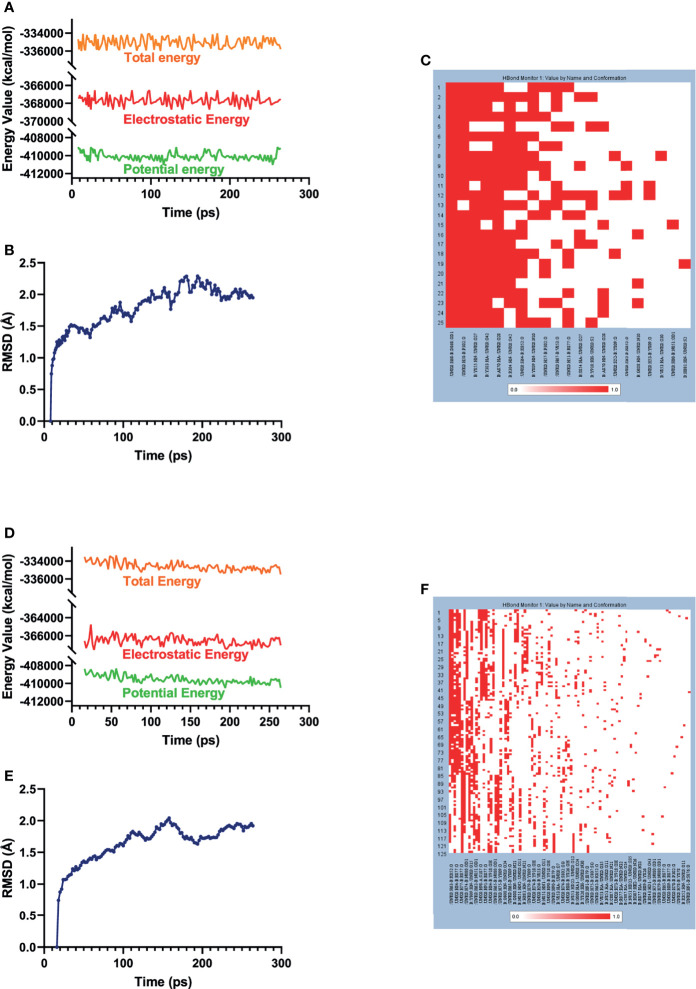
Results of MD simulation of two these complexes. **(A)** Energy values of ZINC000004217536-EZH2 complex during the MD process. EZH2, Enhancer of Zeste Homolog 2; MD, molecular dynamics. **(B)** Average backbone RMSD of ZINC000004217536–EZH2 complex. RMSD, root mean square deviation. **(C)** Chemical bonds heatmap of ZINC000004217536–EZH2 complex in the progression of MD. **(D)** Energy values of ZINC000003938642–EZH2 complex during the MD process. **(E)** Average backbone RMSD of ZINC000003938642–EZH2 complex. **(F)** Chemical bonds heatmap of ZINC000003938642–EZH2 complex in the progression of MD.

The best binding conformations of each compound–EZH2 complexes (ZINC000004217536-EZH2 and ZINC000003938642-EZH2) were obtained from precise docking program CDOCKER and applied for the following experiment. In this study, the stability of the ligand–EZH2 complex under *in vivo* circumstance was predicted by MD simulation module. The predicted results were shown in **Figure 6**, including energy values (**Figures 6A, D**) and RMSD curve (**Figures 6B, E**) diagram of each complex. The orbitals of all complexes reached equilibrium after 100ps. The complexes’ RMSD and energy values like total energy, potential energy, and electrostatic energy all got stabilized over time. Results suggested that hydrogen bonds formed by the compound and EZH2 and the π-π-related interactions contributed a great effect on the stability of these complexes. Furthermore, chemical bonds heatmap after MD also illustrated that these chemical bonds, which contributed largely to the stability of complexes, could still exist steadily with the progression of MD in the body (**Figures 6C, F**). Based on the above evaluation indicators, the complexes formed by ZINC000004217536 and ZINC000003938642 with EZH2 could exist stably in the internal environment. Consequently, these two compounds could interact with EZH2; they also had a regulatory effect on EZH2 like the reference compound GSK126 did.

### ZINC000003938642 Reduced Proliferation of Osteosarcoma Cells

**Figure 7:**
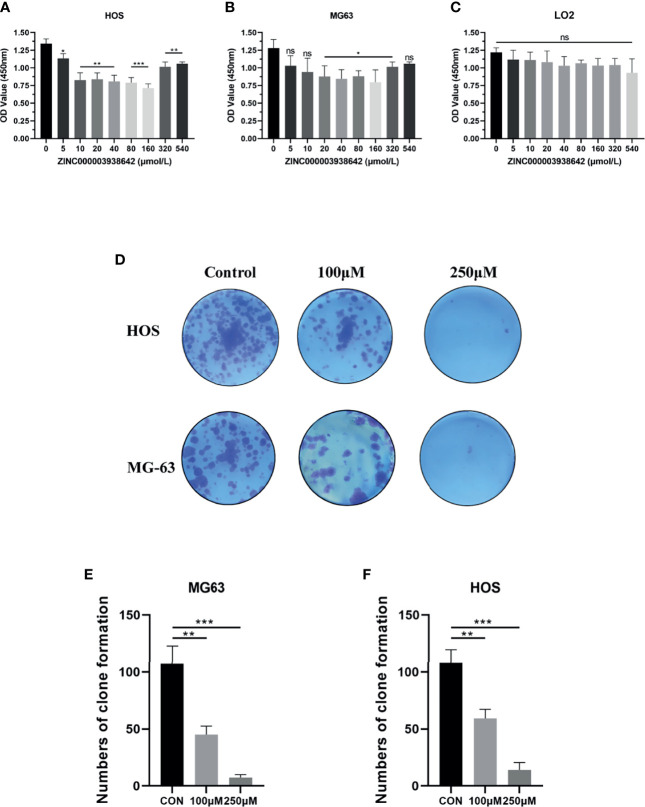
**(A, B)** Cellular viability of osteosarcoma (OS) cell lines (HOS and MG-63) and **(C)** human liver cell line (LO2) treated with different doses of drug ZINC000003938642. **(D)** Clonogenicities in Petri dishes with different doses of drug. **(E, F)** Numbers of clone formation in HOS and MG-63 cell lines. *P < 0.05; **P < 0.01; ***P < 0.0001; ns, none significance.

To test the antitumor effects of compounds screened in this study, we purchased one of the two compounds, ZINC000003938642, for further *in vitro* experiments, aiming to evaluate the effects on OS cells. To assess the proliferation ability of OS cells in the presence of drug ZINC000003938642, the survival of cells after drug treatment was calculated by CCK-8, and growing ability of OS cells was assessed by CFA. The OS cells were treated with different concentrations of drug for 24 h (0, 5, 10, 20, 40, 80, 160, 320, 540 μmol/L). Results indicated that the cellular viability of both HOS and MG-63 cells were declined with the increase of drug concentration (**Figures 7A, B**). Subsequently, to validate the toxicity of drug to liver cells, LO2 cell line was conducted and measured by CCK-8. Results revealed that drug ZINC000003938642 did not inhibit the proliferation of human normal liver cells in a dose-dependent manner and time-dependent manner, which still had a high cellular viability even when subjected to the highest dose (**Figure 7C**).

We then performed CFA to further determine the antitumor effects of drug in OS cells. As shown in **Figure 7D**, after 10 days of cultivation with different drug concentrations (100 and 250 μmol/L), both HOS and MG-63 cells showed fewer and smaller clonogenicities in Petri dishes with drug group than with the control group. The numbers of clone formation in drug groups were significantly lower than those in control groups (P < 0.05) (**Figures 7E, F**).

### ZINC000003938642 Inhibited Migration of Osteosarcoma Cells

**Figure 8:**
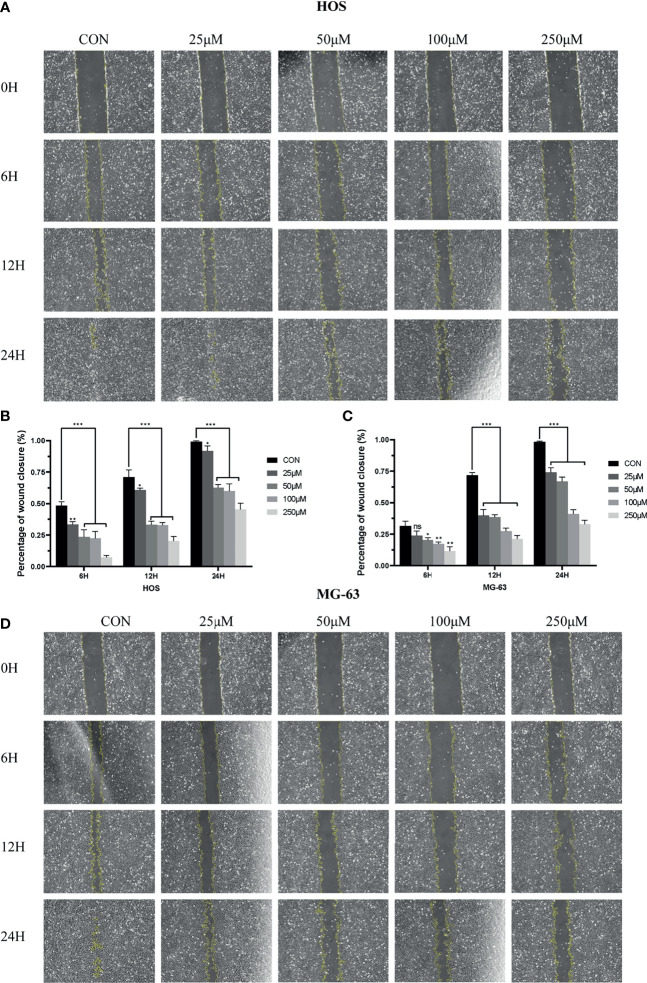
**(A)** Scratch assay of HOS cell line in control and different drug groups at 0, 6, 12, 24 h. **(B)** Percentage of wound closure of HOS cell line in control and different drug groups at 0, 6, 12, and 24 h. **(C)** Scratch assay of MG-63 cell line in control and different drug groups at 0, 6, 12, and 24 h. **(D)** Percentage of wound closure of MG-63 cell line in control and different drug groups at 0, 6, 12, and 24 h. *P < 0.05; **P < 0.01; ***P < 0.0001; ns, none significance.

To analyze the effects of drug ZINC000003938642 on OS cell migration, scratch assay was further performed. The width of scratched areas was measured at 0, 6, 12, and 24 h of scratch, and the scratch width represented the migration capacity of OS cells (**Figures 8A, D**). As shown in **Figures 8B, C**, results exposed that with the extension of time and increase of drug concentration, the migration rate of OS cells to the scratch area decreased significantly (P < 0.05).

### ZINC000003938642 Induced Apoptosis in Osteosarcoma Cells

**Figure 9:**
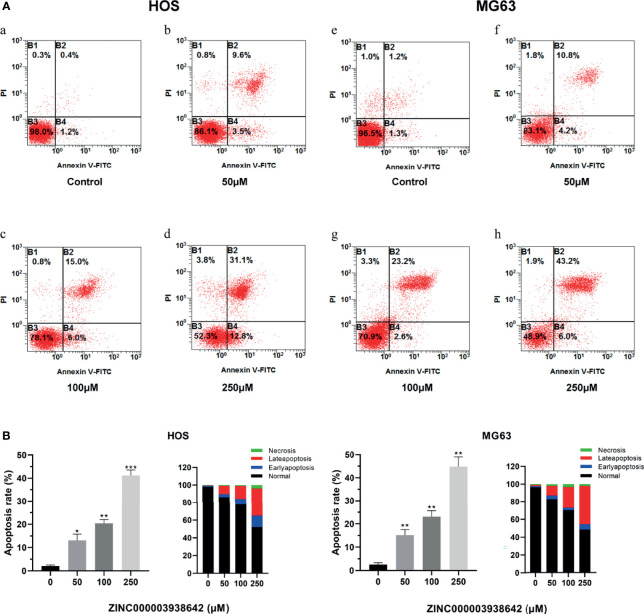
**(A)** The distribution in apoptosis with different concentrations in HOS and MG-63 cells. a–d: drug treatment with 0, 50, 100, and 250μM for 24 h in HOS cells; e–h: drug treatment with 0, 50, 100, and 250 μM for 24 h in MG-63 cells. **(B)** Apoptotic rates and percentage with different concentrations in HOS and MG-63 cells. *P < 0.05; **P < 0.01; ***P < 0.0001.

Flow cytometry and Annexin-FITC/PI double staining were used for apoptosis assay to detect the effects of drugs on programmed cell death. The apoptotic rates of HOS and MG-63 cells were detected after being treated with concentrations of ZINC000003938642 (0, 50, 100, and 250 μM) for 24 h. As shown in **Figure 9A**, results illustrated that the apoptotic rates increased with the increase of drug concentration both in HOS and MG-63 cells. Consequently, live cells were predominant in the control (0 μM) groups, while apoptotic cells were predominant in the drug-treated groups (**Figure 9B**; P < 0.05).

### ZINC000003938642 Reduced the Expression of EZH2 and Its Downstream Gene C-Myc

**Figure 10:**
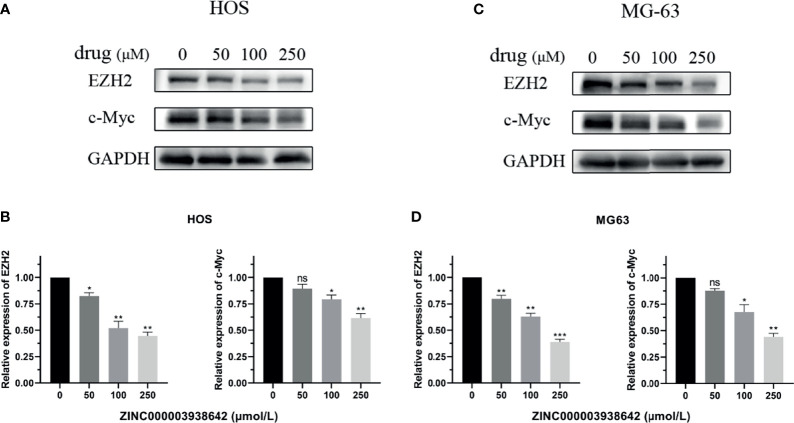
**(A, B)** The expression of Enhancer of Zeste Homolog 2 (EZH2) and its downstream gene c-Myc when treated with different doses of drug in HOS cells. **(C, D)** The expression of EZH2 and its downstream gene c-Myc when treated with different doses of drug in MG-63 cells. *P < 0.05; **P < 0.01; ***P < 0.0001; ns, none significance.

The expression of EZH2 was detected by Western blot analysis. As shown in **Figures 10A, C**, the expression level of EZH2 was inhibited by ZINC000003938642 in both HOS and MG-63 cells, and its inhibitory effect displayed a dose-dependent manner (P < 0.05; **Figures 10B, D**). c-Myc was the downstream target gene of EZH2, and as an oncogene, the expression levels of c-Myc were also reduced by ZINC000003938642 in both HOS and MG-63 cells (**Figures 10A, C**), and its inhibitory effects also demonstrated a dose-dependent manner (**Figures 10B, D**).

### Ligand Pharmacophore Predictions

**Figure 11:**
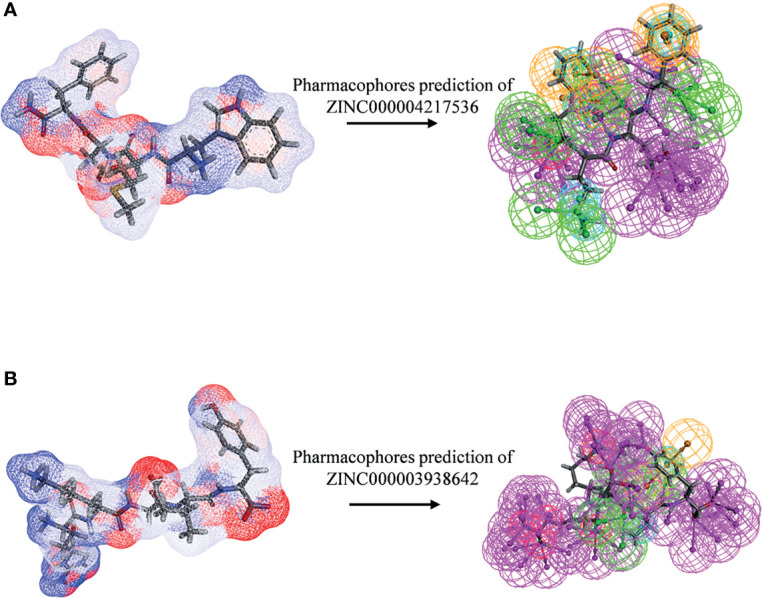
Pharmacophore predictions of **(A)** ZINC000004217536 and **(B)** ZINC000003938642 using 3D-QSAR. Green represents hydrogen acceptor, blue represents hydrophobic center, purple represents hydrogen donor, and orange represents aromatic ring.

After initially verifying the antitumor effects of the candidate compounds, this study further analyzed the pharmacophore characteristics of these two compounds in order to observe the potential modification site on compounds. As shown in **Figures 11A, B**, computational results illustrated that there were 51 features in ZINC000004217536 and 69 features in ZINC000003938642, among which, ZINC000004217536 possessed 18 hydrogen bond acceptors, 21 hydrogen bond donors, five hydrophobic centers, one ionizable positive, and one ring aromatic. As for ZINC000003938642, it possessed 22 hydrogen bond acceptors, 38 hydrogen bond donors, two hydrophobic centers, five ionizable positive, and two ring aromatics.

## Discussion

Statistics published in these years show that malignant tumors are still the main causes of death among residents in many countries (31). OS is one of the most common malignant tumors of mesenchymal tissues, occurring mostly in the metaphysis of long bones in adolescents; it is a differentiation-defective disease caused by dysdifferentiation of osteoblasts and/or epigenetic changes (32). The harm of malignant tumors to humans is not only a threat to the lives of patients but also the physical pain, mental pressure, and the economic burden they bring to patients (33). EZH2, an essential component of the epigenetic regulatory factor PcG and a catalytic subunit of PRC2, is involved in regulating the methylation of lysine 27 (H3K27) of histone H3 and is highly expressed in a variety of tumors. It plays an important role in regulating gene transcription and gene silencing and participates in the growth, proliferation, and metastasis of tumor cells.

In recent years, EZH2 has become a popular target for cancer therapeutics, and the research of EZH2 inhibitors and their combined application with other antitumor drugs in clinical practice has broad prospects (34). However, relatively few inhibitors of EZH2 had been discovered and analyzed. Existing research had reported the high expression of EZH2 in OS patients. Currently, few studies have been conducted on the efficacy of EZH2 inhibitors in OS. GSK126, which is relatively a mature EZH2 inhibitor, was applied in this study to analyze the antitumor effect and molecular mechanism on OS, and it was regarded as the reference compound to compare pharmacologic properties with novel ligands.

Although GSK126 has certain antitumor functions, it still has some limitations. Relevant studies have shown that EZH2 can produce drug resistance through allelic mutations and protein conformation changes (35, 36). For the purpose of overcoming the drug resistance of EZH2, it is necessary to develop new inhibitors. Furthermore, GSK126 had low solubility and rapid plasma clearance, resulting in low bioavailability, and GSK126 had hepatotoxicity, which leads to unsatisfactory effects of high-dose GSK126 in the process of inhibiting EZH2.

In this study, we aimed to discover more potential lead compounds of EZH2. The available natural compound structures were downloaded from the ZINC15 database for virtual screening, then ADME, TOPKAT, CDOCKER, and other modules were applied to perform ADME prediction, rodent carcinogenicity and Ames mutation prediction, ligand–receptor binding studies, and MD simulations. The LibDock scores suggested the degree of energy optimization and conformational stability between compound and receptor. Compounds with higher LibDock scores illustrated better energy optimization and more stable conformation than compounds with lower scores. Calculation results of the LibDock module showed that a total of 13,537 compounds could be stably combined with EZH2 after fast docking method. Among these ligands, 669 compounds had higher LibDock scores than the reference compound GSK126 (LibDock score: 132.143), suggesting that the stability and energy optimization effect of the complex substances formed by these 669 compounds with EZH2 were more stable than GSK126–EZH2 complex. Based on ranking of the LibDock scores, the top 20 compounds with the highest scores were screened out and tested in next steps.

ADME and toxicity prediction were performed to evaluate the pharmacological properties of these selected compounds. After analysis, results elucidated that ZINC000004217536 and ZINC000003938642 had satisfactory intestinal absorption capacity, and these two compounds had no obvious inhibitory effect on CYP2D6, no hepatotoxicity, and low binding affinity property with plasma protein, which suggested the good selectivity of these drugs; they could avoid rapid clearance by plasma so as to behave the best pesticide effect. Furthermore, compared with other compounds, ZINC000004217536 and ZINC000003938642 were predicted not to have Ames mutagenicity and rodent carcinogenicity, and they had less developmental toxicity potential. Consequently, they were considered as ideal candidate compounds with pharmacologic properties and higher security in the body; these characteristics were enough to be considered as the most potential lead compounds. Based on the above results, ZINC000004217536 and ZINC000003938642 were reasonably recognized as high-quality medicinal materials; these two compounds had broad application prospects in drug development and design. Although other drugs on the list had certain negative effects such as developmental toxicity and Ames mutagenicity, other pharmacological properties were relatively moderate, so they also had a certain potential in drug improvement, which could be achieved by adding or deleting specific functional groups or atoms to reduce their negative effects. In summary, it was determined that ZINC000004217536 and ZINC000003938642 were the most potential lead compounds, and more analyses were further performed.

Subsequently, we analyzed chemical bonds and the binding mechanisms between candidate compounds, GSK126 and EZH2. Precise docking method CDOCKER module was conducted; results showed that the CDOCKER interaction energy of ZINC000004217536 and ZINC000003938642 was significantly lower than that of the reference ligand GSK126, proving that the affinity of these two compounds with EZH2 was higher than GSK126–EZH2 in real situations. After that, through molecular detection analysis of their chemical structures among these complexes, results illustrated that the chemical bond and interaction force of the complex formed by EZH2 and the two candidate compounds were stronger, which further explained that they may have a competitive inhibitory effect on the regulatory site of EZH2 and thus inhibit the activity of EZH2, finally producing antitumor effects.

Ultimately, MD simulations were performed to predict the stability of the complexes formed by the candidate compounds and EZH2 in the internal environment. By calculating the RMSD and energy values of these ligand–EZH2 complexes, the RMSD curve and energy curve were drawn. Results showed that the trajectories of the complexes reached equilibrium after 100ps, and the RMSD and energy values of these complexes tended to be stable over time, indicating that the two complexes could exist stably in the internal environment. Furthermore, chemical bonds heatmap elucidated that these chemical bonds, which contributed remarkably to the stability of the complex, could keep steady with the progression of the MD. Consequently, the compounds selected in this study bonded tightly to EZH2, and they were capable of existing stably in the body, thereby exerting corresponding pharmacological functions. Therefore, they have great potential in the development of EZH2 inhibitors. It is noteworthy that the reference compound GSK126 chosen in this study served as a known effective synthetic EZH2 inhibitor; the effects of natural compounds were hardly better than GSK126 *in vitro* or *in vivo*. The role GSK126 played in this process was to provide a primitive crystal complex for us to compare the binding mode and give us an initial active binding sphere, and it did not provide any guiding significance for the comparison between GSK126 and ZINC000003938642 in antitumor aspects.

Currently, existing studies pointed out EZH2 could serve as a therapeutic target regarding OS (11), while few studies focused on targeted therapy of OS targeting EZH2. Consequently, this study preliminarily discussed the effects of newly found compounds against OS. To prove the pesticide effects of our newly found compounds against OS and the reliability of the screening method in this study, we selected one of the candidate compounds, ZINC000003938642, and performed a series of *in vitro* experiments including CCK-8, CFA, scratch assay, Western blot, and apoptosis assay. In CCK-8 assay, results pointed that the cellular viability in OS cells had a dose-dependent decrease when treated with drug ZINC000003938642, while the drug was relatively well tolerated for human liver cells LO2. This finding implied that this drug was relatively nontoxic in term of hepatotoxicity, which was also consistent with our predictions in structural biology part that ZINC000003938642 was a nontoxic drug. In CFA, the numbers and size of clonogenicities in drug group were significantly less than those in control group in both HOS and MG-63 cell lines, which was consistent with results that the proliferation of OS cells was reduced by drug in CCK-8 assay and that the effects were dose-dependent. Scratch assay revealed that the wound area in control group decreased more sharply than that in drug group with time. As for apoptosis assay, flow cytometry results visualized that the percentage of apoptotic cells increased with the drug increasing, the apoptotic rates of HOS and MG-63 cells treated with high drug dose groups were significantly higher than those of control (0 μM) group (P < 0.05). Western blot analysis revealed that EZH2 expression decreased with increasing drug concentrations. Since c-Myc is the downstream target of EZH2, and as an oncogene (37–39), the expression level of c-Myc could also reflect the inhibitory effects of the drug on EZH2. Results displayed that the downstream oncogene c-Myc was also inhibited by the drug in a dose-dependent manner, implying that drug ZINC000003938642 could serve as a potential EZH2 inhibitor. These experiments suggested the ability of drug to inhibit the proliferation, migration, and EZH2 and c-Myc expression of OS cells, which indicated that drug ZINC000003938642 found in this study was an effective inhibitor regarding OS, and EZH2 was a therapeutic target against OS.

The screening of ideal lead compounds is a key step in drug design and development. Regarding the pharmacophore predictions of ZINC000004217536 and ZINC000003938642, they possessed a number of pharmacophores, which elucidated that based on these skeletons of these two compounds, the modification and refinement of the drug could be conducted to further make a whole new design. The natural compounds discovered in this study are of great significance in the development of EZH2 inhibitors. This study provided evidence for the targeted treatment of OS regarding EZH2 and may have the potential to provide better methods for tumor treatment. Besides, in the field of pharmacology, more research could be studied like modifying the molecular structure of the drugs to reduce the toxicity and mutation to continuously improve the pharmacological effect of the inhibitor.

## Conclusions

This study used a series of virtual screening techniques and discovered two natural compounds, ZINC000004217536 and ZINC000003938642, which have the function of inhibiting the active subunit EZH2 of PRC2. These two compounds bind tightly to the target protein. Additionally, they have no carcinogenicity and toxicity, so they can be regarded as potential EZH2 inhibitors. *In vitro* experiments confirmed that drug ZINC000003938642 could inhibit the proliferation and migration of OS, which could serve as potential lead compounds. This study not only provided the pharmacological properties of candidate drugs but also provided meaningful materials for further research of EZH2-targeted inhibitors.

## Data Availability Statement

The original contributions presented in the study are included in the article/supplementary material. Further inquiries can be directed to the corresponding authors.

## Author Contributions

This study was completed with teamwork. Conceived the idea: MY, LW, CY, and WL. Wrote the main article: WL, ZD, and YLZ. Used the software: WL, YLZ, YJZ, HZ, RX, and YY. Downloaded and collected data: WL, ZD, MC, YLZ, DW, and SZ. Analyzed the data: WL, ZD, YLZ, MJ, KL, LW, and CY. Prepared figures: YLZ, WL, ZD, YJZ, MJ, KL, HZ, and SZ. Redressed the article: all authors. Reviewed the article: MY, LW, CY, and WL. All authors contributed to the article and approved the submitted version.

## Funding

This study was supported by a grant from the National Natural Science Foundation of China (No. 82072475).

## Conflict of Interest

The authors declare that the research was conducted in the absence of any commercial or financial relationships that could be construed as a potential conflict of interest.

## Publisher’s Note

All claims expressed in this article are solely those of the authors and do not necessarily represent those of their affiliated organizations, or those of the publisher, the editors and the reviewers. Any product that may be evaluated in this article, or claim that may be made by its manufacturer, is not guaranteed or endorsed by the publisher.

## References

[1] Czermin B Melfi R McCabe D Seitz V Imhof A Pirrotta V. Drosophila Enhancer of Zeste/ESC Complexes Have a Histone H3 Methyltransferase Activity That Marks Chromosomal Polycomb Sites. Cell (2002) 111(2):185–96. doi: 10.1016/s0092-8674(02)00975-3 12408863

[2] Brykczynska U Hisano M Erkek S Ramos L Oakeley EJ Roloff TC. Repressive and Active Histone Methylation Mark Distinct Promoters in Human and Mouse Spermatozoa. Nat Struct Mol Biol (2010) 17(6):679–U47. doi: 10.1038/nsmb.1821 20473313

[3] Kamminga LM Bystrykh LV Boer AC Houwer S Douma J Weersing E. The Polycomb Group Gene Ezh2 Prevents Hematopoietic Stem Cell Exhaustion. Blood (2006) 107(5):2170–9. doi: 10.1182/blood-2005-09-3585 PMC1895717 16293602

[4] Beguelin W Popovic R Teater M Jiang YW Bunting KL Rosen M. EZH2 Is Required for Germinal Center Formation and Somatic EZH2 Mutations Promote Lymphoid Transformation. Cancer Cell (2013) 23(5):677–92. doi: 10.1016/j.ccr.2013.04.011 PMC3681809 23680150

[5] Chase A Cross NCP. Aberrations of EZH2 in Cancer. Clin Cancer Res (2011) 17(9):2613–8. doi: 10.1158/1078-0432.Ccr-10-2156 21367748

[6] Zhu J Jin L Zhang AL Gao P Dai GC Xu M. Coexpression Analysis of the EZH2 Gene Using The Cancer Genome Atlas and Oncomine Databases Identifies Coexpressed Genes Involved in Biological Networks in Breast Cancer, Glioblastoma, and Prostate Cancer. Med Sci Monitor (2020) 26:12. doi: 10.12659/msm.922346 PMC7320634 32595202

[7] Wu SC Zhang Y. Cyclin-Dependent Kinase 1 (CDK1)-Mediated Phosphorylation of Enhancer of Zeste 2 (Ezh2) Regulates Its Stability. J Biol Chem (2011) 286(32):28511–9. doi: 10.1074/jbc.M111.240515 PMC3151093 21659531

[8] Wan LX Xu KX Wei YK Zhang JF Han T Fry C. Phosphorylation of EZH2 by AMPK Suppresses PRC2 Methyltransferase Activity and Oncogenic Function. Mol Cell (2018) 69(2):279–+. doi: 10.1016/j.molcel.2017.12.024 PMC5777296 29351847

[9] He LL Liao LF Du LZ. miR-144-3p Inhibits Tumor Cell Growth and Invasion in Oral Squamous Cell Carcinoma Through the Downregulation of the Oncogenic Gene, EZH2. Int J Mol Med (2020) 46(2):828–38. doi: 10.3892/ijmm.2020.4638 PMC7307824 32626925

[10] Kleer CG Cao Q Varambally S Shen R Ota I Tomlins SA. EZH2 is a Marker of Aggressive Breast Cancer and Promotes Neoplastic Transformation of Breast Epithelial Cells. Proc Natl Acad Sci USA (2003) 100(20):11606–11. doi: 10.1073/pnas.1933744100 PMC208805 14500907

[11] Lv Y-F Yan G-N Meng G Zhang X Guo Q-N. Enhancer of Zeste Homolog 2 Silencing Inhibits Tumor Growth and Lung Metastasis in Osteosarcoma. Sci Rep (2015) 5:12999. doi: 10.1038/srep12999 26265454 PMC4533017

[12] Varambally S Dhanasekaran SM Zhou M Barrette TR Kumar-Sinha C Sanda MG. The Polycomb Group Protein EZH2 is Involved in Progression of Prostate Cancer. Nature (2002) 419(6907):624–9. doi: 10.1038/nature01075 12374981

[13] Koppens MAJ Bounova G Cornelissen-Steijger P de Vries N Sansom OJ Wessels LFA. Large Variety in a Panel of Human Colon Cancer Organoids in Response to EZH2 Inhibition. Oncotarget (2016) 7(43):69816–28. doi: 10.18632/oncotarget.12002 PMC5342517 27634879

[14] Zeng D Liu M Pan J. Blocking EZH2 Methylation Transferase Activity by GSK126 Decreases Stem Cell-Like Myeloma Cells. Oncotarget (2017) 8(2):3396–411. doi: 10.18632/oncotarget.13773 PMC5356890 27926488

[15] Liu J-N Ma Z-L Su R-J Huang K-Q. Effect of Enhancer of Zeste Homolog 2 Inhibitor GSK126 on the Proliferation and Apoptosis of Tongue Squamous Cell Carcinoma. Hua xi kou qiang yi xue za zhi = Huaxi kouqiang yixue zazhi = West China J stomatology (2020) 38(5):495–501. doi: 10.7518/hxkq.2020.05.004 PMC7573763 33085231

[16] Weng CJ Li Y Xu D Shi Y Tang H. Specific Cleavage of Mcl-1 by Caspase-3 in Tumor Necrosis Factor-Related Apoptosis-Inducing Ligand (TRAIL)-Induced Apoptosis in Jurkat Leukemia T Cells. J Biol Chem (2005) 280(11):10491–500. doi: 10.1074/jbc.M412819200 15637055

[17] Shi B Liang J Yang X Wang Y Zhao Y Wu H. Integration of Estrogen and Wnt Signaling Circuits by the Polycomb Group Protein EZH2 in Breast Cancer Cells. Mol Cell Biol (2007) 27(14):5105–19. doi: 10.1128/mcb.00162-07 PMC1951944 17502350

[18] Pereira ER Frudd K Awad W Hendershot LM. Endoplasmic Reticulum (ER) Stress and Hypoxia Response Pathways Interact to Potentiate Hypoxia-Inducible Factor 1 (HIF-1) Transcriptional Activity on Targets Like Vascular Endothelial Growth Factor (VEGF). J Biol Chem (2014) 289(6):3352–64. doi: 10.1074/jbc.M113.507194 PMC3916539 24347168

[19] Ferrara N. Vascular Endothelial Growth Factor. Arteriosclerosis thrombosis Vasc Biol (2009) 29(6):789–91. doi: 10.1161/atvbaha.108.179663 19164810

[20] Lin W Chen Y Zeng L Ying R Zhu F. Effect of a Novel EZH2 Inhibitor GSK126 on Prostate Cancer Cells. Zhejiang da xue xue bao Yi xue ban = J Zhejiang Univ Med Sci (2016) 45(4):356–63. doi: 10.3785/j.issn.1008-9292.2016.07.05 PMC10396985 27868408

[21] Liu SS Rong GH Li X Geng LJ Zeng ZN Jiang DX. Diosgenin and GSK126 Produce Synergistic Effects on Epithelial-Mesenchymal Transition in Gastric Cancer Cells by Mediating Ezh2 *via* the Rho/ROCK Signaling Pathway. OncoTargets Ther (2020) 13:5057–67. doi: 10.2147/ott/s237474 PMC7292386 32606728

[22] Otvos RA Still KBM Somsen GW Smit AB Kool J. Drug Discovery on Natural Products: From Ion Channels to Nachrs, From Nature to Libraries, From Analytics to Assays. SLAS Discov (2019) 24(3):362–85. doi: 10.1177/2472555218822098 PMC6484542 30682257

[23] Li W Yuan B Zhao Y Lu T Zhang S Ding Z. Transcriptome Profiling Reveals Target in Primary Myelofibrosis Together With Structural Biology Study on Novel Natural Inhibitors Regarding JAK2. Aging (Albany NY) (2021) 13(6):8248–75. doi: 10.18632/aging.202635 PMC8034969 33686952

[24] Newman DJ Cragg GM. Natural Products As Sources of New Drugs Over the 30 Years From 1981 to 2010. J Nat Prod (2012) 75(3):311–35. doi: 10.1021/np200906s PMC3721181 22316239

[25] Li W Ding Z Wang D Li C Pan Y Zhao Y. Ten-Gene Signature Reveals the Significance of Clinical Prognosis and Immuno-Correlation of Osteosarcoma and Study on Novel Skeleton Inhibitors Regarding MMP9. Cancer Cell Int (2021) 21(1):377. doi: 10.1186/s12935-021-02041-4 34261456 PMC8281696

[26] Rao SN Head MS Kulkarni A LaLonde JM. Validation Studies of the Site-Directed Docking Program LibDock. J Chem Inf Model (2007) 47(6):2159–71. doi: 10.1021/ci6004299 17985863

[27] Kumar A Yoluk O MacKerell AD Jr. FFParam: Standalone Package for CHARMM Additive and Drude Polarizable Force Field Parametrization of Small Molecules. J Comput Chem (2020) 41(9):958–70. doi: 10.1002/jcc.26138 PMC7323454 31886576

[28] Ke YY Coumar MS Shiao HY Wang WC Chen CW Song JS. Ligand Efficiency Based Approach for Efficient Virtual Screening of Compound Libraries. Eur J medicinal Chem (2014) 83:226–35. doi: 10.1016/j.ejmech.2014.06.029 24960626

[29] Yang L Li W Zhao Y Zhong S Wang X Jiang S. Computational Study of Novel Natural Inhibitors Targeting O(6)-Methylguanine-DNA Methyltransferase. World Neurosurg (2019) 130:e294–306. doi: 10.1016/j.wneu.2019.05.264 31203065

[30] Vire E Brenner C Deplus R Blanchon L Fraga M Didelot C. The Polycomb Group Protein EZH2 Directly Controls DNA Methylation. Nature (2006) 439(7078):871–4. doi: 10.1038/nature04431 16357870

[31] Sung H Ferlay J Siegel RL Laversanne M Soerjomataram I Jemal A. Global Cancer Statistics 2020: GLOBOCAN Estimates of Incidence and Mortality Worldwide for 36 Cancers in 185 Countries. CA-Cancer J Clin (2021) 71(3):209–49. doi: 10.3322/caac.21660 33538338

[32] Xiong XF Zhang JL Liang WG Cao WJ Qin SN Dai LB. Fuse-Binding Protein 1 is a Target of the EZH2 Inhibitor GSK343, in Osteosarcoma Cells. Int J Oncol (2016) 49(2):623–8. doi: 10.3892/ijo.2016.3541 27278257

[33] Roos WP Thomas AD Kaina B. DNA Damage and the Balance Between Survival and Death in Cancer Biology. Nat Rev Cancer (2016) 16(1):20–33. doi: 10.1038/nrc.2015.2 26678314

[34] Kim KH Roberts CWM. Targeting EZH2 in Cancer. Nat Med (2016) 22(2):128–34. doi: 10.1038/nm.4036 PMC4918227 26845405

[35] Gibaja V Shen F Harari J Korn J Ruddy D Saenz-Vash V. Development of Secondary Mutations in Wild-Type and Mutant EZH2 Alleles Cooperates to Confer Resistance to EZH2 Inhibitors. Oncogene (2016) 35(5):558–66. doi: 10.1038/onc.2015.114 PMC4744243 25893294

[36] Baker T Nerle S Pritchard J Zhao B Rivera VM Garner A. Acquisition of a Single EZH2 D1 Domain Mutation Confers Acquired Resistance to EZH2-Targeted Inhibitors. Oncotarget (2015) 6(32):32646–55. doi: 10.18632/oncotarget.5066 PMC4741719 26360609

[37] Koh CM Iwata T Zheng Q Bethel C Yegnasubramanian S De Marzo AM. Myc Enforces Overexpression of EZH2 in Early Prostatic Neoplasia *via* Transcriptional and Post-Transcriptional Mechanisms. Oncotarget (2011) 2(9):669–83. doi: 10.18632/oncotarget.327 PMC3248223 21941025

[38] Zhang X Zhao X Fiskus W Lin J Lwin T Rao R. Coordinated Silencing of MYC-Mediated miR-29 by HDAC3 and EZH2 as a Therapeutic Target of Histone Modification in Aggressive B-Cell Lymphomas. Cancer Cell (2012) 22(4):506–23. doi: 10.1016/j.ccr.2012.09.003 PMC3973134 23079660

[39] He L Weber A Levens D. Nuclear Targeting Determinants of the Far Upstream Element Binding Protein, a C-Myc Transcription Factor. Nucleic Acids Res (2000) 28(22):4558–65. doi: 10.1093/nar/28.22.4558 PMC113884 11071946

